# Accelerated idioventricular rhythm as a manifestation of chronic renocardiac syndrome: A case report

**DOI:** 10.1111/anec.13131

**Published:** 2024-06-25

**Authors:** Kotzadamis Dimitrios, Gkroumtsia Evangelia, Papadopoulos Christodoulos, Vassilikos Vassilios

**Affiliations:** ^1^ Third Department of Cardiology Hippokration General Hospital of Thessaloniki Thessaloniki Greece; ^2^ Department of Nephrology Papageorgiou General Hospital Thessaloniki Greece

**Keywords:** accelerated idioventricular rhythm, advanced chronic kidney disease, hemodialysis, uremic toxins

## Abstract

In this case report, we describe a patient who presented with chronic symptoms and signs of uremia and persistent accelerated idioventricular rhythm (AIVR) on electrocardiogram. Findings from blood tests, echocardiography, renal ultrasound, and renal scan were suggestive of heart failure with reduced ejection fraction and chronic kidney disease, and attendance of daily hemodialysis sessions led to the restoration of sinus rhythm. Typically, AIVR has a favorable prognosis and, if necessary, medical intervention focuses on addressing the underlying responsible causes. Accumulation of uremic toxins has the potential to trigger the formation of AIVR and clearance of small solutes through conventional hemodialysis may contribute to sinus rhythm restoration.

## Graphical abstract

Accelerated idioventricular rhythm in a non‐dialysis patient with advanced chronic kidney disease terminated by hemodialysis.

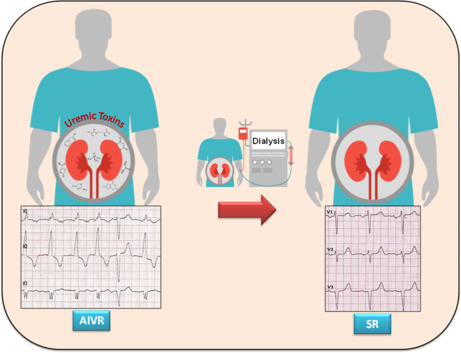

## 1 INTRODUCTION

Accelerated idioventricular rhythm (AIVR) is an uncommon arrhythmia, notable mainly for indicating successful reperfusion after acute myocardial infarction. However, it has been observed in patients with drug intoxications, cardiomyopathies, electrolyte disturbances, and in healthy individuals as well (Riera et al., 2010). Herein, we report a case of persistent AIVR in a non‐dialysis patient with advanced chronic kidney disease (CKD) terminated by initiation of hemodialysis.

## 2 CASE PRESENTATION

A 50‐year‐old White man with inadequate medical treatment due to loss to follow‐up presented to his primary care physician complaining of itchy skin and easy fatigability. He had a past medical history of ischemic heart disease, type 2 diabetes mellitus, and chronic kidney disease, and he was on regular furosemide 250 mg, bisoprolol 10 mg, allopurinol 100 mg, aspirin 100 mg, linagliptin 5 mg, and insulin glargine 14 IU daily. A 12‐lead electrocardiogram (ECG) was performed that showed AIVR. The patient was referred to the emergency department (ED), but he was reluctant to do so. Finally, he visited the ED 3 days later of his own volition.

**FIGURE 1:**
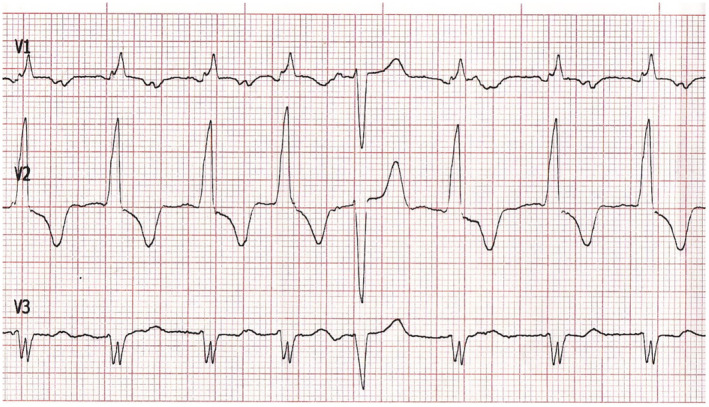
ECG tracing on admission showing AIVR and a capture beat.

On admission to the ED, the patient was alert and oriented, in no acute distress and had normal vital signs. A repeat ECG confirmed the presence of AIVR with 90 ventricular beats per minute and scarce capture beats (Figure 1). Physical examination revealed normal heart sounds with a mild systolic murmur while thoracic auscultation was unremarkable. Mild pitting edema of both lower extremities with nonhealing skin ulcers with serosanguinous drainage were present, aggravated by intense pruritus. Moreover, he conceded chronic poor appetite and loss of weight despite fluid retention.

Blood tests were remarkable for severely impaired kidney function (urea 286 mg/dL, creatinine 4 mg/dL, 2021 CKD‐EPI eGFR 17 mL/min/1.73 m^2^), iron deficiency anemia (hemoglobin 11 g/dL, ferritin 67.8 ng/mL, transferrin saturation 12%), hypoalbuminemia (albumin 3.1 g/dL), and elevated brain natriuretic peptide (BNP 3283 pg/mL), while electrolytes, liver, and thyroid function tests, arterial blood gas, cardiac enzymes, and inflammatory markers were all within normal range (Table 1). A spot urine sample revealed no albuminuria.

**TABLE 1:** Patient's basic metabolic panel.

	3 months before admission	Day 1 admission	Day 2	Day 3	Day 4	Day 5 discharge
Glucose (mg/dL)	103	155	98	80	87	118
Urea (mg/dL)	258	286	223	158	130	129
Creatinine (mg/dL)	4	4	3.1	2.8	2.7	2.7
Sodium (mmol/L)	134	135	138	138	137	137
Potassium (mmol/L)	3.7	3.9	3.7	3.5	3.9	3.5
Magnesium (mmol/L)	2.3	1.9	2	2	1.9	2
Calcium (mg/dL)	8.7	8.7	8.8	8.7	8.8	8.9
Phosphorus (mmol/L)	4.9	4.5	4.5	4.4	4.5	4.4
Chloride (mmol/L)	100	99	100	99	99	101

Transthoracic echocardiography showed a left ventricle with normal size, severe global hypokinesia, and an estimated ejection fraction of 25%–30%. Right ventricle had normal size but impaired systolic function as well. Moreover, there was mild biatrial enlargement, mild tricuspid, and mitral valve regurgitation and aortic valve sclerosis. Renal ultrasound revealed small sized kidneys, no renal pelvis dilatation, thin and hyperechogenic parenchyma, and loss of corticomedullary differentiation. Furthermore, a ^99m^Tc‐MAG_3_ renal scan was performed that showed decreased renal perfusion, radionuclide uptake, and excretion of both kidneys, while there was no blockage of urine flow.

The patient met multiple criteria that warranted the start of long‐term hemodialysis treatment (sustained and severely reduced eGFR, bleeding diathesis, pruritus, volume overload refractory to high doses of diuretics, deteriorating nutritional status, and persistent fatigue) so a Cardiorenal Interdisciplinary Team meeting was called that favored this decision. Despite the abnormal ECG, there was absence of hemodynamic significance, so a dialysis catheter was placed and he attended conventional intermittent hemodialysis sessions. It is noteworthy that, due to the patient's normal urine output, dialysis sessions were conducted with an emphasis on diffusion with minimal ultrafiltration volume.

**FIGURE 2:**
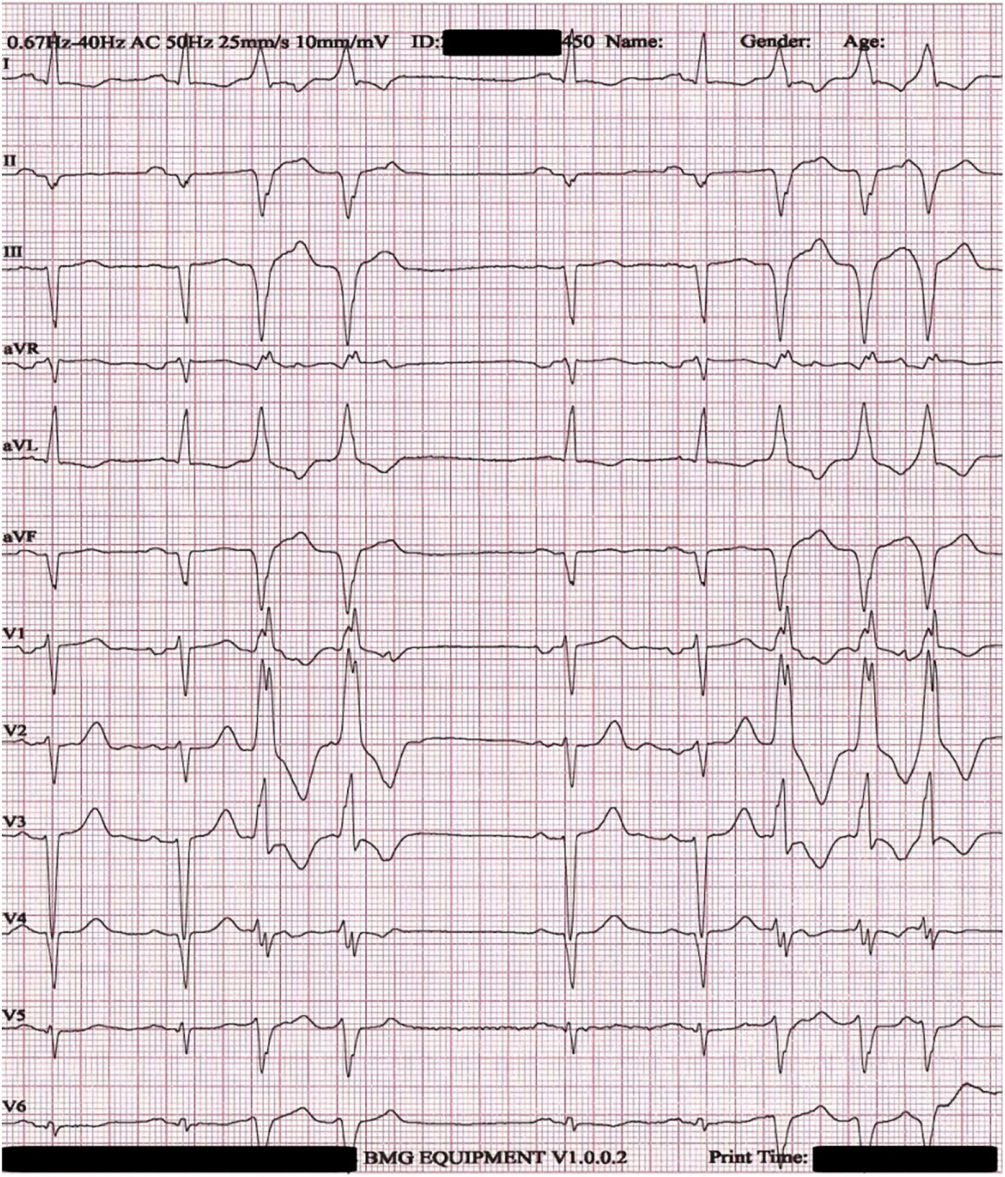
ECG showing sinus rhythm with salvos of premature ventricular complexes on day 3.

**FIGURE 3:**
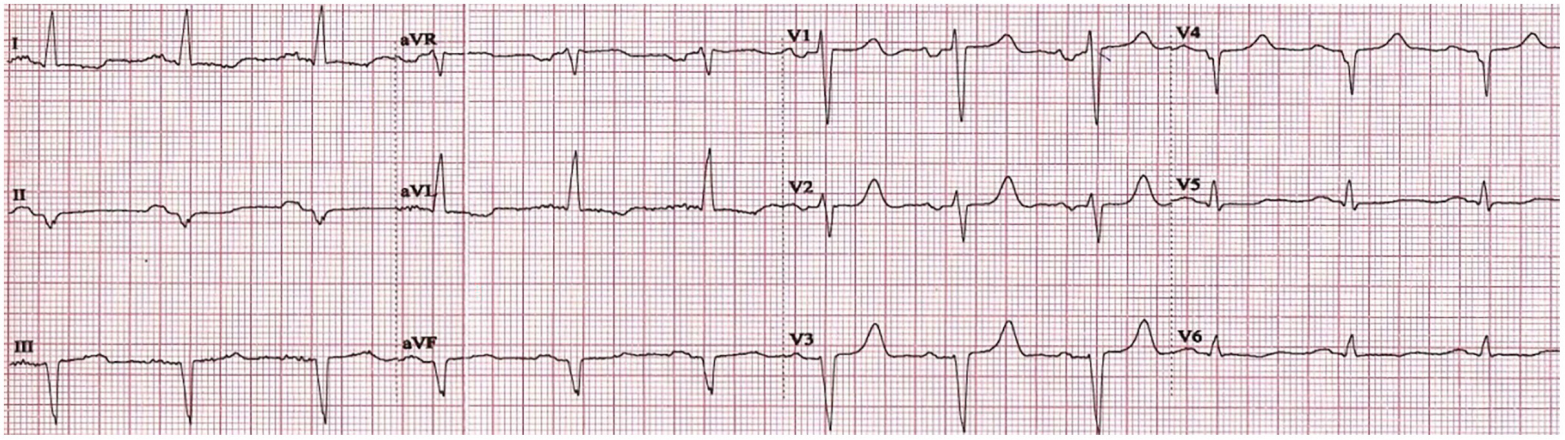
ECG showing complete restoration of sinus rhythm before discharge.

Next day, the patient underwent his second dialysis session while still maintaining AIVR. On Day 3, a new dialysis session was performed, and a new ECG showed restoration of sinus rhythm with salvos of premature ventricular complexes originating from the same ventricular focus (Figure 2). The following day, there was complete restoration of sinus rhythm on ECG (Figure 3). An invasive coronary angiography was performed that revealed a chronic three‐vessel disease. The patient was discussed at the Heart Team meeting, and coronary artery bypass graft surgery was decided as the optimal method of revascularization. On Day 5, the patient had his final dialysis session during his inpatient hospital stay, while maintaining sinus rhythm, and his left ventricular ejection fraction was mildly improved (LVEF 30%–35%). After being introduced to the hospital's hemodialysis schedule, he was discharged with significant clinical improvement.

On follow‐up visit, the patient had complete remission of his symptoms. ECG tracing showed normal sinus rhythm. He had enrolled a hemodialysis program with three sessions per week while being on a waiting list for elective coronary artery bypass surgery. Lastly, an Implantable Cardioverter–Defibrillator implantation was proposed with the purpose of primary prevention.

## 3 DISCUSSION

To the best of our knowledge, it is described for the first time in this report a case of a non‐dialysis patient with advanced CKD who presented with persistent AIVR that was restored to sinus rhythm after initiation of daily hemodialysis sessions.

AIVR is a rare arrhythmia characterized by three or more consecutive beats originating from a ventricular focus at a rate between 50 and 110 bpm. It is most commonly observed after reperfusion of an acute myocardial infarction; however, it has been documented in cases with drug intoxications (e.g., digoxin, beta agonists, and anesthetic agents), electrolyte disturbances, cardiomyopathies (e.g., hypertrophic, dilated, and arrhythmogenic), and in healthy individuals without any underlying cardiac pathology (Riera et al., 2010). It is important to note that typically AIVR has a favorable prognosis and, if necessary, medical intervention focuses on addressing the underlying responsible causes (Bijsterveld et al., 2022). It can be easily postulated that in our case report, chronic uremic state was the trigger for AIVR formation.

It is well‐established that multiple interactions exist between heart and kidneys and cardiovascular disease (CVD) often leads to kidney injury through a cascade of neurohormonal, hemodynamic, and inflammatory mechanisms. These pathophysiological interactions gave birth to the so‐called cardiorenal syndrome (Ronco et al., 2008). Nevertheless, the heart–kidney relationship is bidirectional as CKD is a strong risk factor for adverse cardiovascular events (Kingma Jr. et al., 2015). CKD‐related CVD accounts for almost 50% of all‐cause mortality in CKD patients and manifests in clinical practice mainly as coronary artery disease, stroke, heart failure, and arrhythmias (Tonelli et al., 2016). Regarding the latter, CKD patients are vulnerable to heart rhythm disorders and exhibit an increased burden of supraventricular (especially atrial fibrillation) and ventricular tachyarrhythmias, and sudden cardiac death (SCD) (Turakhia et al., 2018).

The probability of SCD is inversely related to eGFR, but SCD is a leading cause of death even in young patients in the early stages of CKD, suggesting mechanisms other than ischemia. Increased predilection for ventricular arrhythmias and SCD in CKD patients seems to stem from complex processes closely related to metabolic disarrangements secondary to renal dysfunction, in contrary to individuals with normal kidney function in which acute coronary syndrome, systolic heart failure, and myocardial scars are the usual pathological substrate (Bonato & Canziani, 2017; Tonelli et al., 2016).

In particular, uremic solutes that are inadequately excreted or metabolized due to diminished renal function, but remain biologically active, are termed uremic toxins (UTs). UTs tend to accumulate with subsequent catastrophic consequences on virtually all organ systems. Based on their physicochemical properties and the ability of conventional hemodialysis to remove them, UTs are most commonly classified into small UTs (molecular weight ≤500 Da), middle UTs (molecular weight >500 Da), and protein‐bound UTs (Moradi et al., 2013). Whereas small UTs are freely filtered, a great proportion of middle and protein‐bound UTs are difficult to remove via conventional low‐flux hemodialysis membranes and their gradual build‐up is considered a strong non‐traditional risk factor for CVD in hemodialysis‐dependent patients (Moradi et al., 2013). Actually, considerable emphasis has been placed on unfiltered compounds such as fibroblastic growth factor‐23, indoxyl sulfate, and p‐cresyl sulfate. In pre‐dialysis stages, however, the accumulation of small UTs exerts harmful biological activity on the myocardium as well (Lekawanvijit, 2018). This is clearly highlighted in our case report in which small UTs clearance through the first hemodialysis sessions led to an immediate reduction of arrhythmia burden, putting a halt to the vicious cycle of renocardiac syndrome.

Enhanced automaticity and triggered activity initiated by afterdepolarizations in ventricular fibers are two mechanisms that are most consistent with AIVR formation in a vulnerable myocardium under the cumulative impact of small UTs (Antzelevitch & Burashnikov, 2011; Hsueh et al., 2014). Arrhythmia could allegedly be either considered a coincidental finding or attributed to ischemic heart disease. Still, the fact that sinus rhythm recovery was achieved through diffusive dialysis sessions with low ultrafiltration volume, while no other intervention was made, suggests that the uremic milieu is responsible.

There is a crucial knowledge gap in the understanding of the codependent relationship between the heart and the kidneys. This gap becomes more evident in regard to arrhythmias formation and SCD in CKD patients (Boriani et al., 2015). While their role is increasingly recognized, the involvement of UTs, especially of those with low molecular weight, in cardiac electrophysiological remodeling and the underlying molecular mechanisms are incompletely understood and need to be further evaluated in experimental studies (van Ham et al., 2022). Advances in hemodialysis techniques by using all available membrane separation processes and introducing novel dialysis membranes that allow the clearance of middle, large, and even protein‐bound UTs will also assist in this direction.

## AUTHOR CONTRIBUTIONS

KD conception, initial draft and completion of manuscript. GE, PC and VV review and revisions to manuscript.

## CONFLICT OF INTEREST STATEMENT

There is no conflict of interest.

## ETHICS STATEMENT

All the procedures were conducted according to the principles of the Helsinki Declaration.

## CONSENT

Written informed consent has been obtained from the patient in line with COPE guidelines.

## Data Availability

The data that support the findings of this study are available from the corresponding author upon reasonable request.
